# Case report: Chronological symptom profile after cessation of overdose zolpidem in a patient with comorbid bipolar disorder—from anxiety, craving, paresthesia and influenza-like symptoms to seizures and hallucinations

**DOI:** 10.3389/fpsyt.2022.962836

**Published:** 2022-08-17

**Authors:** Zi-xin Mao, Xia Yang, Hui-yao Wang, Wan-jun Guo

**Affiliations:** ^1^Mental Health Center and Psychiatric Laboratory, West China Hospital, Sichuan University, Chengdu, China; ^2^Department of Neurobiology, Affiliated Mental Health Center and Hangzhou Seventh People’s Hospital, Zhejiang University School of Medicine, Hangzhou, China

**Keywords:** zolpidem, withdrawal symptoms, overdose, chronological feature, bipolar disorder

## Abstract

**Introduction:**

Insomnia is a major public health problem that determines the quality of life. Among the many causes of insomnia, psychological factors have an important influence on the process, duration of insomnia, help-seeking behavior, and treatment choice. Regarding medical treatment, zolpidem is always chosen to treat acute and transient insomnia due to its few side effects. Although some randomized controlled trials have verified its safety, zolpidem abuse and withdrawal reactions have been reported in recent years.

**Case report:**

A 25-year-old unmarried man with a college degree who worked as a graphic designer was referred and admitted to the inpatient ward for a chief complaint of “alternative episodes of lowering and elevation of mood for 10 years, overdosage use of zolpidem for two years.” He underwent a time-dependent withdrawal reaction after admission. It was characterized by rebound insomnia, anxiety, craving, skin paresthesia, influenza-like symptoms, tonic-clonic-type seizures, and hallucinations. At the 1-year follow-up, he did not exhibit any remaining withdrawal symptoms.

**Discussion:**

The acute cessation of overdosage zolpidem use causes a series of withdrawal symptoms that manifest in chronological order. Additionally, long-term benzodiazepine exposure has potential influences on zolpidem dependence/tolerance. However, patients with a history of abuse or dependence, or mental disorders seem to be at risk of drug abuse. Clinicians should be alert to the potential for zolpidem dependence and addiction. Once the acute cessation of overdosage zolpidem use occurs, the potential of the withdrawal reaction needs to be considered and addressed properly.

## Introduction

As a highly prevalent determinant of quality of life, insomnia has become a major public health concern. It was reported that more than half of the people surveyed had experienced insomnia at least once a month (1). Even in surveys using stringent diagnostic criteria, the prevalence of insomnia disorder is reported to be approximately 10% (1–3). The course of insomnia can be chronic and persist for many years (2–4). In addition to being an independent diagnosis, insomnia is also common comorbidity of various psychiatric/neurological disorders, such as mood disorders and anxiety disorders, that require adequate therapy (2, 4–7).

There are many causes of insomnia. Among them, psychological factors have an important influence not only on the process and duration of insomnia but also on help-seeking behavior and treatment choices, including increments or reductions in drug dosage (8). The most frequently reported psycho-social factors that lead to insomnia are stressful events, while the cognitive characteristics of high expectations of sleep duration and excessive worry of sleep loss and sleep distortion may significantly affect the automaticity of the sleep-wake system and drug dosage management (9, 10), which “reminds” people to rely on drugs and increases the dosage used during self-healing and even produces “craving” to maintain addiction (8).

Zolpidem (Stilnox^®^) is an imidazopyridine that is different from benzodiazepines in chemical structure. It is commonly chosen to treat acute and short-term insomnia due to its rapid onset, short duration of action, and few side effects (11). Zolpidem has been used for decades in the clinic. In 1992, it was approved for use by the U.S. Food and Drug Administration (FDA) (12).

Theoretically, zolpidem has some advantages over benzodiazepines in terms of the receptors it binds to, adverse reactions, and pharmacokinetics (13–15). At the recommended dose, zolpidem acts on the GABA_*A*_ α_1_ subunit selectively and has a sedative effect. However, at high doses, it loses selectivity. It also acts on α_2_, α_3_, and α_5_ subunits and causes muscle relaxation, antianxiety and anticonvulsant effects (11, 13). After a single oral dose of 5–10 mg of zolpidem, the peak plasma concentrations (*t*_*max*_) were found to occur in a range from 0.5 to 2 h (16), and the half-life (*t*_1/2_) ranged from 2.5 to 2.6 h (15). Thus, zolpidem acts quickly, have a short half-life, and has nearly no significant residual effect the next day (17).

Since zolpidem was marketed, some clinical trials have verified the safety and efficacy of 10 mg of zolpidem for acute and transient insomnia disorder. Studies found no obvious signs of rebound insomnia or withdrawal symptoms after 28 or 180 days of treatment (18–21). Over the past two decades, there have been some case reports regarding the withdrawal symptoms following the cessation of overdose zolpidem use, especially seizures (the summary of reports is presented in Table 1) (22–29). The authors of previous reports focused on one of the serious or typical withdrawal symptoms but did not observe the different kinds of zolpidem withdrawal symptoms in one case, nor the symptom profile over time.

**TABLE 1:** Studies reviewed for withdrawal symptoms of zolpidem.

References	Age	Sex	Day does (mg)	Duration of zolpidem use (months)	Time of zolpidem withdrawal (hours)	Psychiatric diagnosis	Withdrawal Symptoms
Aragona (22)	43	Female	400–600	24	4	Insomnia	Generalized tonic-clonic seizures
Barrero-Hernández et al. (23)	50	Female	450	Several months	12	1. Chronic insomnia 2. Anxiety	Seizures
Cubała and Landowski (24)	29	Female	160	24	12	1. Dissociative disorder 2. Insomnia	Generalized tonic-clonic seizures
Oulis et al. (25)	49	Female	1,500	48	Not mentioned	1. Dysthymic disorder 2. Insomnia	Consecutive seizures of partial epilepsy (right upper limb)
Haji Seyed Javadi et al. (26)	30	Female	100–150	3	16	1. Dysthymic disorder 2. Chronic insomnia	Generalized tonic-clonic seizures
Castillo (27)	26	Male	50–100	12	24	Bipolar disorder	Generalized tonic-clonic seizures
Hadinezhad and Hosseini (28)	32	Female	100	9	24	Major depressive disorder	Epileptic attack
Russo et al. (29)	27	Male	600	Several years	24	1. Fractured tibia 2. Insomnia	Epileptic attack
Baruch et al. (30)	41	Female	45	36	24	1. Major depressive disorder 2. Dissociative identity disorder 3. Substance abuse 4. Insomnia	Agitation, weakness, and remains intractable nausea
Huang et al. (31)	34	Female	1,000 2,000	24 3		1. Zolpidem dependence and withdrawal 2. Dysthymic disorder	Anxiety, cold sweating, hand tremor, sweating, palpitations, distractible attention and impaired memory, generalized tonic-clonic seizure
Jana et al. (32)	33	Male	100–120 (max 150)	36	Skipping a dose or delaying	1. Insomnia 2. Nicotine dependence	Anxiety, restlessness, apprehension of something unpleasant, a low mood, lack of enthusiasm or interest in work, impairment in concentration
Chen et al. (33)	59	Female	1. High dose of different BZD and hypnotics	Many years	Not mentioned	1. Bipolar disorder 2. Benzodiazepines dependence	Insomnia, anxiety, restlessness, and hand tremors
			2. Zolpidem 600 mg	24			
Spyridi et al. (34)	78	Male	300	60	24	1. Tolerance, abuse, and dependence on benzodiazepines 2. Insomnia	Anxiety, irritability, and insomnia and incapable of managing everyday difficulties
Keuroghlian et al. (35)	34	Male	100–150 200	30 2	72	1. Bipolar disorder 2. Alcohol abuse 3. Prescription opioid abuse 4. Sleep cycle disturbances	Generalized tonic-clonic seizures, blurred vision, headache, anxiety, and “jitters”
Heydari and Isfeedvajani (36)	32	Male	400	6	A few days	1. Opioid dependent 2. History of tic and anxiety	Restlessness, irritability, runny nose, diarrhea, sweating, palpitation, insomnia, myalgia, muscle cramps, muscle tic, and jump
Pourshams and Malakouti (37)	62	Female	570	24	Not mentioned	1. Zolpidem withdrawal syndrome 2. Major depressive disorder 3. Opium dependency	Agitation, crying, anxiety, impatience, loss of energy, insomnia, irritability, verbal aggression, distraction, increased appetite, physical symptoms (headaches and lightheadedness), shivering, and craving
Bajaj et al. (38)	45	Male	2,400	60	6–8 h	1. Alcohol dependence 2. Zolpidem dependence and withdrawal	Weakness of limbs, inability to concentrate, light headedness, severe insomnia, tremors, and marked irritability
Kar and Dwivedi (39)	56	Male	70–100	2	2–3 h	1. Bipolar affective disorder 2. Epilepsy 3. Insomnia	Aggressive, restless, oddities behavior, increased psychomotor activity, irrelevant talk, and burning sensation in the scalp
Ravishankar and Carnwath (40)	55	Female	200	30	168	1. Depression 2. Insomnia	Low mood, disturbed sleep, nightmares, sweating, tremors, panic attacks, and episodes of confusion
	28	Male	100	Several years	2 weeks (reduction to 40 mg)	Not mentioned	Anxiety, panic attacks, and fear of going outside
Chiaro et al. (41)	70	Female	1,200	Not mentioned	Not mentioned	1. Tension-type headache 2. Primary insomnia	A striking fragmentation of the sleep structure, a severe reduction of sleep duration, sleep efficiency and slow wave sleep, and an increased REM sleep latency

We present a case of chronological symptoms that occurred after overdose zolpidem withdrawal to help clinicians recognize this process early and address it effectively.

## Case report

A 25-year-old unmarried man with a college degree who worked as a graphic designer was referred and admitted to the inpatient ward for a chief complaint of “alternative episodes of the lowering and elevation mood for 10 years, overdosage use of zolpidem for two years.”

The patient had a history of psychiatric diseases. Eleven years prior to admission, after experiencing sadness, low energy, hopelessness, and insomnia for half a year, he was diagnosed with *depressive disorder* in a local hospital. The patient was treated with 20 mg of fluoxetine per day and 10 mg of zolpidem every night. Depression and insomnia were gradually alleviated in 1 year. He reported that he had taken the same drugs and dosage for another 6 years, but symptoms fluctuated during this time. Seven years prior to admission, at the beginning of college, he experienced an episode of elevated mood characterized by the feeling of being in a good condition, increased ability, and high energy levels, and he remained busy for a long time. Five years prior to admission, during the third year of college, when he experienced stress regarding his studies and problems in a closed relationship, he experienced another depressive episode characterized by sadness, loss of interest, low energy, hopelessness, and insomnia for half a year. During this episode, he continued to take the same drugs as those he was first prescribed. When the condition gradually improved, he stopped taking zolpidem and fluoxetine. Four years prior to admission, during the fourth year of college, he experienced a second episode of elevated mood characterized by the feeling of increased energy levels and ability and increased interest and participation in many activities. This period of elevated mood lasted for nearly one and a half years, during which he had driven 20,000 km to travel around the United States. He denied taking drugs during this episode. Two years prior to admission, when he began his first job as a graphic designer, another depressive episode characterized by sadness, loss of interest, low energy, hopelessness, and insomnia started. He reported that he felt similarly to how he felt during the episode 11 years ago. He resumed taking zolpidem 10 mg per night and fluoxetine 20 mg per day. Sometimes he took another 10 mg of zolpidem on his own for a better hypnotic effect. Five months before visiting, his work got busier and his stress levels increased, and he found that taking zolpidem could make him feel relaxed. Thus, the patient took additional zolpidem before bedtime until he relaxed. In total, he took a maximum of 80 mg of zolpidem once or twice weekly, with an average of 30 mg every night. His family members observed that he performed some abnormal and dangerous behaviors after taking an overdose of zolpidem at night, including cheerfulness, talkativeness, frequent movement, excessive eating, and dangerous driving behaviors associated with several traffic accidents. To prevent similar dangerous behaviors and improve sleep and mood, his father brought him to the hospital for help. To improve the possibility of successful drug withdrawal for a patient with comorbid bipolar disorder, insomnia, and long-term zolpidem dependence who repeatedly performed high-risk behaviors and had a higher possibility of severe withdrawal reactions once the drug was stopped, the patient was hospitalized in the psychiatric inpatient ward in the West China Hospital of Sichuan University.

In addition, he had a history of alcohol abuse and cigarette smoking, but he reported that he had given up alcohol and smoking 4 years prior to admission. He admitted that he occasionally used marijuana and nitrous oxide during college, but he denied any use of those drugs for at least 1 year, which was confirmed by his father. He also denied any recent use of alcohol and other sedative-hypnotic drugs, which was confirmed by his father. He had no family history of mental illness.

On admission, the patient was 176 cm tall and 86 kg in weight, and his vital signs were in the normal range. Physical and neurological examinations revealed no notable abnormalities. ECG, electroencephalogram (EEG), chest CT, and brain MRI yielded normal findings. Laboratory tests revealed alanine aminotransferase levels of 62 IU/L, aspartate aminotransferase levels of 30 IU/L, creatinine levels of 65 μmol/L, and an estimated glomerular filtration rate (eGFR) of 128.54 ml/min/1.73 m^2^. The complete blood count (CBC), electrolyte levels, cortisol levels, adrenotropin levels, and thyroid function were in the normal range. The blood concentration of lithium carbonate was 0.055 mmol/L on the seventh day.

According to the 10th revision of the International Classification of Diseases (ICD-10), he was diagnosed with *“Harmful use of sedative or hypnotic drugs*; *bipolar affective disorder, current episode moderate depressive*.” The treatment strategies used for zolpidem withdrawal followed the protocols used in previous case reports (26, 29, 31–34, 37–39, 41, 42). The treatment for comorbid bipolar disorder followed the guidelines for the management of bipolar disorder (43).

**FIGURE 1:**
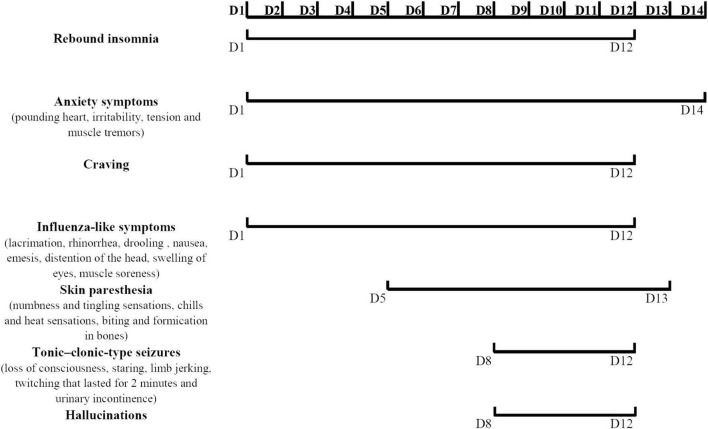
Symptom-time chart.

**FIGURE 2:**
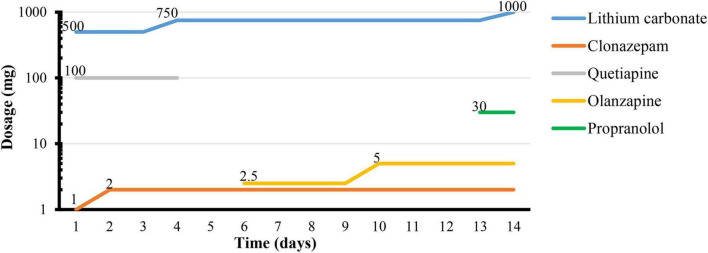
Details of treatment.

After zolpidem cessation, a series of symptoms appeared gradually during hospitalization. Zolpidem use was terminated the first night, and the patient described obvious rebound insomnia, influenza-like symptoms (manifested as distension of the head, swelling of eyes, and muscle soreness), anxiety symptoms (manifested as muscle tension and muscle tremors) accompanied by strong craving. He was treated with clonazepam (1 mg every night), lithium carbonate (250 mg twice daily), and quetiapine (100 mg every night). On the third day, rebound insomnia became serious, and he could not sleep until 4:00 a.m. Influenza-like symptoms and anxiety symptoms (manifested as pounding heart, irritability, tension, and muscle tremors) persisted during each attack. The attacks lasted for approximately 20 min from onset to symptom relief. The clonazepam dosage was increased to 2 mg per night, and the lithium carbonate dosage was increased to 250 mg three times daily. On the fifth day, skin paresthesia (manifested as numbness and tingling sensations, chills and heat sensations, and biting and formication in bones) appeared and worsened in the following days. The possibility of skin paresthesia that may be caused by quetiapine was considered. This drug was replaced with 2.5 mg olanzapine every night. On the sixth day, he experienced typical influenza-like symptoms, such as lacrimation, rhinorrhea, drooling, nausea, and emesis. The duration of each attack increased to more than 30 min from onset to relief. On the eighth to tenth days, the symptoms of some attacks appeared in the following order: first, anxiety symptoms; then, a mixture of skin paresthesias and influenza-like symptoms; then, loss of consciousness, staring, limb jerking, and twitching that lasted approximately 2 min; then, hallucination and urinary incontinence followed the relief of limb twitching and return to consciousness. The patient drew what he saw after symptom relief. The symptoms of loss of consciousness, staring, limb jerking, twitching that lasted for 2 min, and urinary incontinence were considered to be the onset of tonic-clonic-type seizures. However, the EEG was not obtained during the attack. Similar tonic-clonic-type seizures occurred on average once a day during this stage. Craving was also readily reported during this stage. The duration from the onset of anxiety symptoms to the relief of tonic-clonic-type seizures and subsequent urinary incontinence and hallucination lasted not more than 40 min. Tonic-clonic-type seizures were considered one of the withdrawal symptoms, and diazepam was prepared as necessary. He was given 5 mg olanzapine in total to control the hallucinations. On the 12th day, the occurrence of tonic-clonic-type seizures, hallucinations, and cravings decreased. The duration of each symptomatic attack from onset to relief was reduced to approximately 10 min. After 12 days of hospitalization, only the anxiety symptoms (manifested as palpitations and worry) and skin paresthesia (manifested as tingling and formication) appeared occasionally. The duration of each symptomatic attack was reduced to no more than 5 min. Propranolol (10 mg three times daily) was added to the regimen to relieve palpitations. On the 14th day, most of the withdrawal symptoms were relieved except anxiety. The patient was discharged after 15 days. The details of the symptom changes over time are shown in Figure 1, and the drug treatment plan is shown in Figure 2.

After discharge, the patient was followed up in the outpatient department. Three months after discharge, the lithium carbonate dosage was reduced to 250 mg three times daily, the clonazepam dosage was 1 mg every night, and the olanzapine and propranolol dosages were not changed. In the fourth month after discharge, he experienced several anxiety attacks characterized by nervousness, sweating, and trembling that lasted approximately 10 min with spontaneous remission. There were no anxiety attacks reported in the fifth month of follow-up. The olanzapine, propranolol, and clonazepam dosages were reduced gradually. He did not report any skin paresthesia, seizure, or hallucination after discharge. He was sleepless and anxious occasionally, but he could cope with it, and he never took zolpidem again. Finally, 12 months after discharge, he took 250 mg of lithium carbonate twice daily and 0.25 mg of clonazepam every night. He reported that his life and work were normal. This finding confirms that the chronological symptoms observed during hospitalization were most likely caused by zolpidem withdrawal.

## Discussion

To our knowledge, this report represents the first case of chronological symptoms after overdose zolpidem withdrawal. From the data collected, the lowest dosage that induced withdrawal symptoms was 45 mg (Table 1). Symptoms may appear within 2 h after withdrawal, but the duration from symptom onset to relief is not mentioned. It is well known that zolpidem can lead to withdrawal symptoms if overdosed. The details of symptoms were not complete in previous case reports, but they were described in chronological order in our case report. It could be assumed that the withdrawal symptoms did not appear randomly. This inference was related to the timing of the cessation of drug intake. The knowledge provided by this study may help to clinically diagnose and treat zolpidem withdrawal in different stages.

The patient with a long history of fluoxetine use with zolpidem may have had an influence on the symptoms that appeared during zolpidem withdrawal due to drug-drug interactive effects and the acute cessation of fluoxetine use. A case report indicated that the combination of zolpidem and fluoxetine might cause hallucinations (44). Studies of the interaction between zolpidem and fluoxetine show that the time of zolpidem onset may be shortened in the presence of fluoxetine (45–47). To some extent, fluoxetine may prolong the duration of zolpidem-associated hallucinations (47). Both zolpidem and fluoxetine are highly protein bound. The interaction between them may occur through competitive binding and increase the serum levels of zolpidem (48). However, some symptoms that occur during zolpidem withdrawal as shown in case reports and clinical trials, such as nervousness, insomnia, dizziness, headache, drowsiness, tremor, and sweating, have also been reported as fluoxetine-related adverse effects (49–51), but those adverse symptoms tended to occur in the early stage of fluoxetine use and infrequently occur during long-term treatment (51). The abrupt interruption of fluoxetine use may cause dizziness and sleep disturbances, but the severity of the discontinuation syndrome is slight, and the score of discontinuation emergent signs and symptoms (DESS) is lower than that of other selective serotonin reuptake inhibitors (SSRIs) (52). This might be because fluoxetine and norfluoxetine have metabolites with longer half-lives, and the blood concentration does not drop quickly. Thus, most symptoms that occurred during zolpidem withdrawal, in this case, were not attributable to the long-term use or abrupt interruption of fluoxetine treatment.

Clinicians should not ignore the potential for zolpidem dependence and addiction because it has high receptor selectivity. Although the number of pharmacological studies on the dependence/tolerance of zolpidem is very limited, it is likely appropriate to interpret its potential mechanisms based on studies of benzodiazepine tolerance solely because the GABA receptor is the major targeted receptor for both zolpidem and benzodiazepines. There are five potential mechanisms underlying the influences of long-term benzodiazepine exposure (53, 54). First, a change in GABA_*A*_ receptor gene expression, which might be related to the exposure time (55), might result in the development of tolerance, although most reports suggested that the number of GABA_*A*_ receptors might not change because of the maximal binding capacity (B_*max*_) of [^3^H] flunitrazepam (a non-selective benzodiazepine) did not change significantly during the studied periods (54). Second, the changes in the subunit composition of GABA_*A*_ receptors might influence the binding of drugs to receptors, especially any changes in the expression of γ submit or α submit (53, 54). Third, uncoupling of the GABA and benzodiazepine binding sites may reduce benzodiazepine activity (53, 54). Vlainić et al. documented that the number of functional interactions occurring at GABA_*A*_ receptors in the zolpidem group was diminished by approximately 40% of the control values (54). Fourth, the phosphorylation of the GABA_*A*_ submits might regulate the components of GABA_*A*_ receptors and the effects of allosteric modulators and consequently influences the function of GABA_*A*_ receptors (53, 54). Fifth, the internalization of GABA_*A*_ receptors, which might be controlled by phosphorylation or by the mediation of uncoupling, may decrease the number of receptors at the cell surface (53, 54), although it probably does not influence the total number of GABA_*A*_ receptors. More pharmacological studies are needed to confirm these potential mechanisms.

Patients with a history of abuse or dependence or mental disorders are potentially at risk for drug abuse (56). It is important to manage the use of benzodiazepines in those patients (57). As shown in this report and others, in addition to insomnia, these patients can exhibit comorbid mental disorders, such as dysthymic disorder, bipolar disorder, alcohol abuse, and so on (Table 1). Patients reported that zolpidem use not only helped with sleep but also relieved anxiety and promoted relaxation (32, 38). Clinicians should be more vigilant about drug abuse and addiction.

This case report had certain limitations. First, there is the possibility that some symptoms of this patient might be caused by substance-related exogenous psychosis (58). It was difficult to rule out that the symptoms were not caused by the history of substance use or the clinical deterioration of bipolar episodes. Second, the urine test which may help distinguish the withdrawal reaction of drugs from intoxication for a patient who had a history of drugs and alcohol abuse, was not complete when the patient was admitted. It is noteworthy that not only the patient but also his father denied that he had recently used alcohol or any other sedative-hypnotic drugs. Third, drug abuse or addiction is a chronic and recurring process, and a systemic follow-up plan of patient monitoring will help evaluate the therapeutic effect and progression of the disease. The time interval of follow-up also needs to be considered.

## Conclusion

The present case report describes the chronological symptom profile of overdose zolpidem withdrawal. It emphasizes the potential of zolpidem abuse and the variability of withdrawal symptoms that change over time. Clinicians should be able to recognize zolpidem early and accurately during the process of zolpidem withdrawal.

## Data availability statement

The original contributions presented in this study are included in the article/supplementary material, further inquiries can be directed to the corresponding author.

## Ethics statement

Written informed consent was obtained from the individual(s) for the publication of any potentially identifiable images or data included in this article.

## Author contributions

Z-XM wrote the first draft. W-JG and H-YW supervised the patient and provided the treatment. W-JG and XY edited the manuscript. All authors contributed to the article and approved the submitted version.
